# Tilting Pad Thrust Bearing Fault Diagnosis Based on Acoustic Emission Signal and Modified Multi-Feature Fusion Convolutional Neural Network

**DOI:** 10.3390/s25030904

**Published:** 2025-02-02

**Authors:** Meijiao Mao, Zhiwen Jiang, Zhifei Tan, Wenqiang Xiao, Guangchao Du

**Affiliations:** 1 School of Mechanical Engineering and Mechanics, Xiangtan University, Xiangtan 411105, China; 2 Engineering Research Center of Complex Trajectory Machining Process and Equipment, Ministry of Education, Xiangtan University, Xiangtan 411105, China

**Keywords:** tilting pad thrust bearing, fault diagnosis, convolutional neural network, acoustic emission signal

## Abstract

Tilting pad thrust bearings are widely utilized in large rotating machinery such as steam turbines and hydraulic turbines. Defects in their shaft tiles directly impact lubrication characteristics, thereby influencing the overall safety performance of the entire unit. To address this issue, this paper presents a fault diagnosis method for tilting pad thrust bearings using a modified multi-feature fused convolutional neural network (MMFCNN). Initially, an experimental bench for diagnosing faults in tilting pad thrust bearings was developed to collect multi-channel acoustic emission (AE) signals from both normal and faulty pads. Subsequently, the squeeze-and-excitation (SE) module was employed to reallocate the weights of each channel and fuse the features of multi-channel signals. Learning was then conducted on the signal fused with multiple features using the inverse-add module and spanning convolution. Next, a comparative analysis was carried out among the CNN1D, ResNet, and DFCNN models, and the MMFCNN model proposed in this study. The results show that under consistent operating conditions, the MMFCNN model achieves an average fault diagnosis accuracy of 99.58% when utilizing AE signal data from tilting pad thrust bearings in four states as inputs. Furthermore, when different operational conditions are introduced, the MMFCNN model also outperforms other models in terms of accuracy.

## 1. Introduction

Tilting pad thrust bearings are commonly found in various high-speed, high-load large-scale equipment such as internal combustion engines, steam turbines, and hydraulic turbines. The operational conditions of these machines are often harsh, highlighting the critical importance of maintaining the proper function of tilting pad thrust bearings to ensure equipment safety and reliability [1,2,3,4]. Acting as a vital support element, the tilting pad thrust bearing plays a central role in the overall machinery operation. Any malfunction in this component can have diverse impacts on machinery performance, potentially resulting in severe mechanical incidents, significant financial losses, and even endangering personal safety [5,6].

Vibration signals serve as effective indicators of machinery condition, offering a combination of convenient measurement techniques and cost efficiency. Consequently, fault diagnosis in rotating machinery utilizing vibration signals has emerged as the most prevalent approach in the field [7,8]. Zhang et al. [9] proposed a novel parametric adaptive variational modal decomposition (VMD) method, leveraging the grasshopper optimization algorithm, for the analysis of vibration signals in rotating machinery, and the results demonstrate that the proposed approach effectively analyzes machinery vibration signals, facilitating accurate fault diagnosis. Yang et al. [10] conducted a comprehensive analysis of vibration signals through time, frequency, and time-frequency domains using the stacked generalization (SG) ensemble model, which demonstrated superior identification accuracy for the Milling tool’s state. Chen et al. [11] proposed a novel fault diagnosis method for gearboxes that integrates complementary ensemble empirical mode decomposition (CEEMD), sample entropy, and correlation analysis algorithms (CorAA). The effectiveness of this approach was validated using data collected from gearboxes operating under various conditions. Yan et al. [12] proposed an intelligent diagnostic method for rolling bearings that integrates symplectic geometry mode decomposition (SGMD), improved multiscale symbolic dynamic entropy (IMSDE) and multiclass relevance vector machine (MRVM), the results show that the method can achieve higher recognition accuracy.

In recent years, the fault diagnosis of rotating machinery based on AE signals has emerged as a central research focus for many scholars [13,14,15,16]. With advancements in the AE mechanism, instrument performance, and signal processing technology, AE testing has become increasingly efficient and effective in recent years [17,18]. In comparison to vibration signals, AE signals offer several advantages, including a wide frequency range of 10 kHz–1000 kHz, the transient elastic wave signals generated by localized energy within the material are less susceptible to interference from ambient noise, and the signals demonstrate high sensitivity for in-situ damage detection [19,20,21,22]. The AE detection technique is a dynamic non-destructive monitoring method that captures signals generated during the early stages of defect development within an object. Brandon et al. [23] introduced a methodology that integrates outlier frequency reduction techniques, time-synchronized resampling, and spectral averaging to process AE signals and derive state indicators for bearing fault diagnosis, experimental results illustrated the method’s effectiveness in diagnosing four fault types, including the inner ring, outer ring, rolling element, and cage faults. He et al. [24] utilized a combination of the short-time root-mean-square function and autocorrelation function to extract the characteristic vibration frequency of bearings, the analysis of AE signals using standard signal parameters demonstrated considerable efficacy in extracting the genuine characteristic frequency of bearings through AE signals. Huang et al. [25] established the relationship between AE parameters and influencing factors of sliding bearings, comparing the characteristics of AE signals and vibration signals under the same conditions, the results indicated that the fault diagnosis method for sliding bearings based on AE signals outperformed vibration signals. Leveraging these advantages, this study employs AE signals for fault diagnosis of tilting pad thrust bearings.

The integration of multi-sensor data has garnered increasing attention from researchers due to its ability to provide richer data and more comprehensive status information. Huang et al. [26] proposed a first-kind flexible tensor singular value decomposition (1K-FTSVD) method in order to address the limitations of the traditional tensor SVD based on the n-mode product, and verified the effectiveness of the method with dynamic simulations signals and experimental data collected from a bearing test rig and a gearbox. Ma et al. [27] proposed a multichannel signal fusion processing method based on flexible tensor singular spectrum decomposition (FTSSD) with adaptive embedding dimension selection to address the difficulty in extracting fault features from dual-channel signals, and the experimental results demonstrate that the method achieves high accuracy and robustness in multichannel signal decomposition and feature extraction. Xu et al. [28] developed a semi-supervised classifier for graph-embedded low-rank tensor learning machine (GE-LRTLM) to implement the extraction of the most important feature and patterns from tensor data while ensuring that the tensor data structure remains unchanged, and the results demonstrate that the method achieves a high classification accuracy while also verifying that the combination of the constructed labeled and unlabeled multi-sensor information fusion tensor samples can promote to the improvement of model accuracy.

Deep learning (DL) has emerged as a crucial research area within machine learning, particularly for its advanced capabilities in processing large datasets. This technology finds extensive applications across various domains, including speech recognition, image analysis, and more. Among the most prevalent networks is the convolutional neural network (CNN) [29]. Guo et al. [30] introduced a novel hierarchical learning rate adaptive deep convolutional neural network (ADCNN), which is built upon an enhanced algorithm, and successfully applied this model to bearing fault diagnosis, and the results demonstrated the model’s rapid training speed and high accuracy. Similarly, Xie et al. [31] developed an improved residual CNN approach that transformed multi-sensor signals into RGB images using principal component analysis prior to inputting them into the network, affirming the model’s effectiveness in diagnosing machine faults under various operating conditions and with multi-sensor datasets. Tang et al. [32] converted hydraulic pump signals into a time-frequency distribution using continuous wavelet transform, and subsequently employed Bayesian optimization for the adaptive learning of hyperparameters in CNN, and the research showed that this method achieved superior accuracy and enhanced robustness. Additionally, Zhao et al. [33] proposed a class-aware adversarial multiwavelet CNN specifically designed for cross-domain fault diagnosis, effectively addressing challenges associated with incomplete feature extraction and the underutilization of unlabeled target data.

In recent years, a number of scholars have made significant contributions to the study of combining AE signals and CNN. Liu et al. [34] proposed a convolutional neural network-based transfer learning (CNN-TL) approach to address the limitations in pipeline leakage detection, which is often confined to complex signal processing techniques and computational resources and only target specific working conditions, and validated it using various AE datasets, the results demonstrate that the proposed CNN-TL model can accurately detect pipeline leaks across a diverse range of operational scenarios. Li et al. [35] proposed a method by integrating CNN and gated recurrent unit (GRU) networks with vibration and AE signals for gear pitting fault diagnosis, and the results showed that the proposed method has higher accuracy and better robustness. Wang et al. [36] proposed a pattern recognition model for pipeline AE online monitoring signals based on blind source separation and CNN to reduce the risk of pipeline failure due to long-term corrosion, and the experimental data showed that the model can be used to accurately identify corrosion signals and has a high overall recognition accuracy. Kim et al. [37] proposed a bearing fault diagnosis technique based on AE signals and CNN, which takes the normalized bearing characteristic component (NBCC) as the input and extracts the important weights using gradient-weighted class activation mapping (Grad-CAM), and experimental results show that the method achieves high classification accuracy with reasonable visualisation. In conclusion, a variety of scholars have made noteworthy contributions to the integration of AE signals with CNN, particularly in applications such as pipeline monitoring, gear failure detection, and bearing diagnostics. Building upon this foundation, the present study leverages AE signals from tilting pad thrust bearing, employing CNN for condition monitoring and fault diagnosis.

While significant advancements have been achieved in mechanical fault diagnosis through DL techniques, there are still some limitations in the existing studies:

1. The majority of studies employing CNN have predominantly focused on the fault diagnosis of gears, gearboxes, rolling bearings, and electric motors. There is a notable lack of research addressing plain bearings, especially tilting pad thrust bearings;
2. Despite the growing emphasis on AE signals and DL techniques, research that integrates AE signals with DL methods for intelligent machinery diagnosis has predominantly concentrated on components such as pipelines, gears, and rolling bearings, with insufficient relevant research applied to the field of plain bearings.

This paper’s principal contributions are as follows:

1. Tilting pad thrust bearings are investigated as subjects of research, utilizing signals that simulate operational scenarios for fault diagnosis;
2. AE signals are employed as a substitute for traditional vibration signals. The raw AE signals are converted into grayscale maps, which serve as inputs for the CNN;
3. An enhanced multi-feature fusion CNN model is proposed, demonstrating recognition accuracy for AE signals derived from tilting pad thrust bearings across various operational conditions.

## 2. Proposed Intelligent Fault Diagnosis Method

AlexNet, a prominent CNN model, demonstrates significant capabilities for classification and recognition tasks due to its automated feature learning processes [38]. In this context, this study proposes the MMFCNN, which builds upon the foundational architecture of AlexNet. The proposed network architecture consists of three distinct feature learning channels. The primary channel integrates the traditional AlexNet framework augmented with an additional pooling layer to enhance feature extraction. Auxiliary Channel 1 introduces an inverse-add module designed for periodic feature learning, while Auxiliary Channel 2 utilizes spanning convolution techniques to effectively capture shallow features. The features extracted from these three channels are subsequently concatenated along the depth dimension following the pooling layer, facilitating a comprehensive representation of the input data.

### 2.1. Construction of the MMFCNN

**Figure 1:**
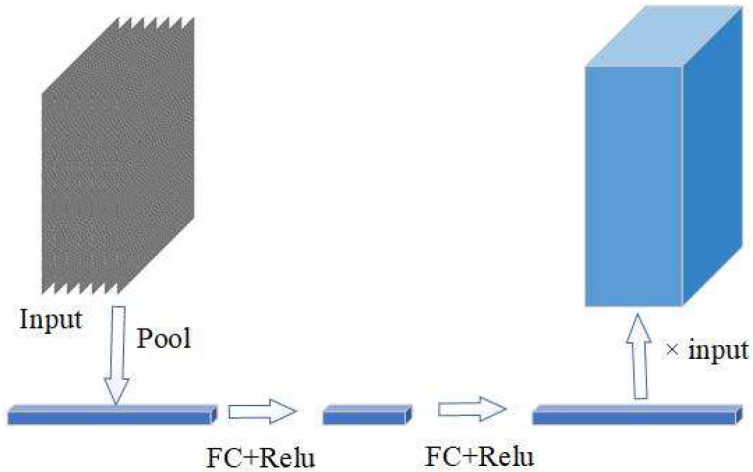
SE module.

To enhance the significance of critical channels while attenuating those of lesser importance, the MMFCNN architecture concurrently transforms signals captured by multiple sensors into grayscale images. These maps are subsequently superimposed to create a comprehensive multi-channel input signal. To effectively weigh the contributions from diverse channels, an SE module is employed. The precise workings of this process are shown in Figure 1. By leveraging this approach, MMFCNN is able to elevate the prominence of essential channels and reduce the influence of less relevant ones, thereby improving the network’s capability to analyze complex multi-sensor data with greater precision.

The MMFCNN processes input data by first converting it into a column vector composed of multiple elements through global pooling. This vector is then transformed into a new column vector via a series of sequential operations that include full joins and activation functions. These operations effectively scale the values down and then back up. Each element in the resulting column vector represents the weight assigned to the various input matrices after this adjustment. These computed weights are subsequently used to multiply the input grayscale image, resulting in the modified input data.

**Figure 2:**
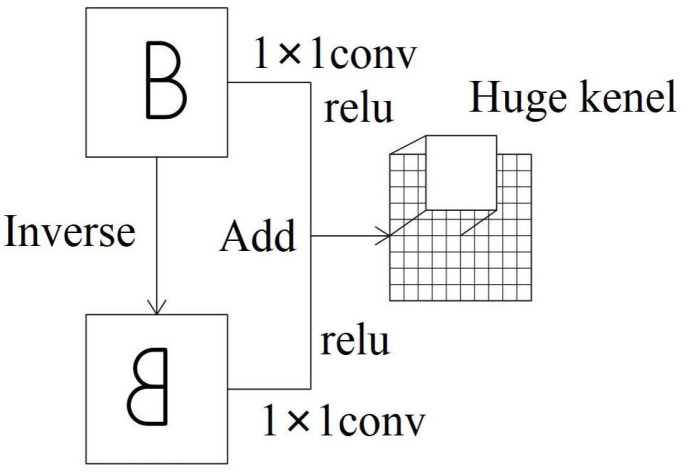
Inverse-add module.

Tilting pad thrust bearings, integral cyclic components in rotating machinery, exhibit distinctive cyclic signaling patterns that reflect their operational state. Deviations in the bearing’s condition manifest as alterations in these cyclic patterns, underscoring the importance of understanding overall cyclic characteristics for accurate condition assessment. Consequently, the learning process necessitates the consolidation of complete cyclic signals. In the subsequent test employing an AE device with a sampling frequency of 1000 kHz, AE signals were collected over one complete revolution of the bearing operating at 2970 RPM and the acquisition resulted in a total of 20,202 data points. However, the conventional convolutional kernels used in image processing are inadequate for capturing the entire bearing cycle within a single learning instance. To address this limitation, the MMFCNN incorporates an inverse-add module. This module utilizes a 1 × 1 convolutional kernel to nonlinearly process and combine the input data with its horizontally flipped counterpart. Following this, cyclic feature extraction is performed on the amalgamated dataset using an expanded convolutional kernel, effectively capturing the complete periodic features inherent in the cyclic signals. The detailed process is shown in Figure 2.

**Figure 3:**
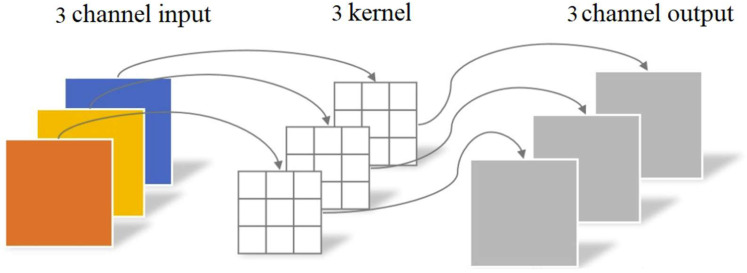
Depth-wise convolution.

The application of large convolutional kernels in CNN is effective in suppressing high-frequency noise. However, this approach often results in a significant increase in the number of parameters, which can hinder the training speed of the model. To address this challenge, the convolution operation within the proposed MMFCNN employs depth-wise convolution. In this method, each convolutional kernel is responsible for processing only a single channel, thereby enhancing computational efficiency. The detailed process is shown in Figure 3.

As the neural network undertakes the task of learning the intricate characteristics of tilting pad thrust bearings, it focuses on key parameters such as amplitude, phase, frequency, and other local attributes that exist between adjacent data points. In the deeper layers of the network, individual neurons synthesize information across the entire sensory field, thereby capturing the holistic characteristics of the input signal. During the failure of tilting pad thrust bearings, the emergence of amplitude modulation (AM), frequency modulation (FM), and phase modulation (PM) phenomena is critical for accurate fault diagnosis. The precise detection and emphasis on signals originating from the impact point are essential for effective fault identification. To address the diagnostic challenges, a series of experiments were conducted utilizing convolutional layers with varying kernel sizes. The experimental results indicate that the network achieves optimal performance with the following configurations: kernel sizes of [11, 2, 113], [5, 4, 57], and [5, 8, 29], accompanied by step sizes of 2, 4, and 8, respectively. Additionally, the number of channels was set to 7, corresponding to the seven acoustic emission sensors employed in the experiment.

### 2.2. Model Parameters

**Figure 4:**
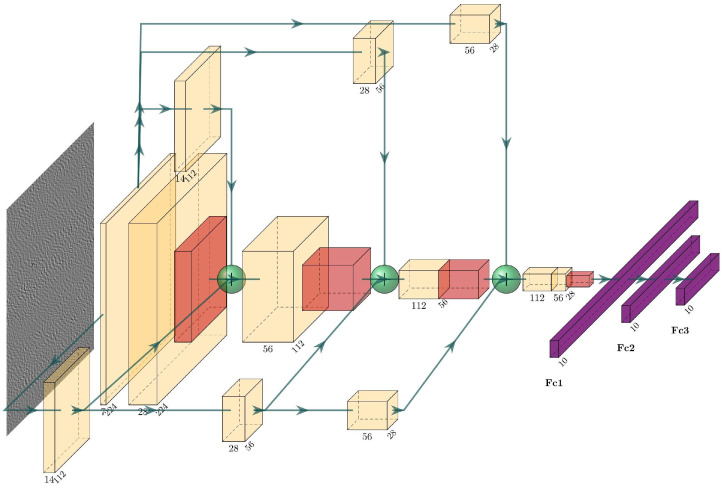
Schematic structure of the MMFCNN.

To effectively manage the dimensionality of the feature matrix prior to the fully connected layer, a maximum pooling layer is incorporated following the third convolutional layer. The input grayscale image has dimensions of 224 × 224 pixels. Specific parameter configurations for the MMFCNN model are presented in Table 1, while the schematic structure of the MMFCNN is shown in Figure 4.

### 2.3. Implementation of the Proposed Method

**Figure 5:**
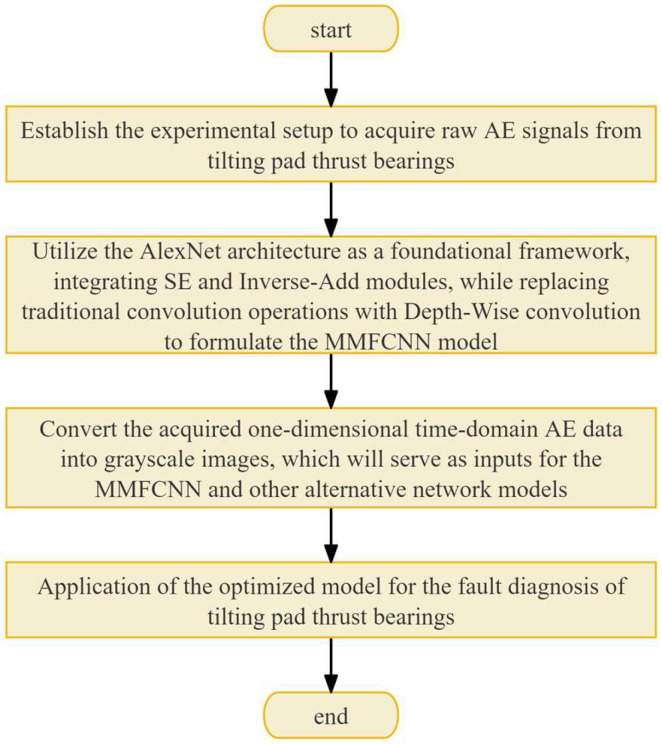
Flowchart of the proposed fault diagnosis method.

The flowchart of the proposed fault diagnosis method is shown in Figure 5 and encompasses the following key steps:

1. Establish the experimental setup to acquire raw AE signals from tilting pad thrust bearings;
2. Utilize the AlexNet architecture as a foundational framework, integrating SE and inverse-add modules, while replacing traditional convolution operations with depth-wise convolution to formulate the MMFCNN model;
3. Convert the acquired one-dimensional time-domain AE data into grayscale images, which will serve as inputs for the MMFCNN and other alternative network models;
4. Application of the optimized model for the fault diagnosis of tilting pad thrust bearings.

## 3. Experimental Data Collection

### 3.1. The Test Bench of the Tilting Pad Thrust Bearing

**Figure 6:**
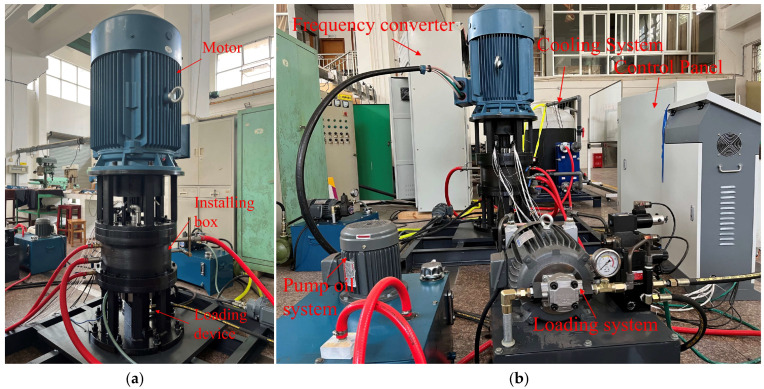
The test bench of the tilting pad thrust bearing: (**a**) The key components of the test bench; (**b**) layout of the test bench.

The experimental data for this research was obtained using a custom-built tilting pad thrust bearing test bench. The key components of the test bench are shown in Figure 6a. The motor, located at the top, is responsible for the rotation of the shaft. The tilting pad thrust bearing is housed within an installing box and is connected to the motor via a motor bracket. The spindle is supported at both ends by rolling bearings, while the loading device, situated at the base of the unit, applies the necessary axial loads. Additionally, Figure 6b shows the layout of the test bench. This bench incorporates several crucial elements, including a cooling system, pumping oil system, loading system, control panel, and frequency converter. Each component is integral to ensuring the smooth operation of the test rig and the reliability and accuracy of data acquisition during the experiments.

### 3.2. Data Acquisition

In this experiment, the tilting pad thrust bearing pads play a pivotal role in determining its performance. To this end, four distinct types of faulty pads—normal, burned, 2 mm scratched, and 3 mm scratched—were acquired to capture AE signals corresponding to different states. The conditions and descriptions of these tilting pad thrust bearing pads are detailed in Table 2.

**Figure 7:**
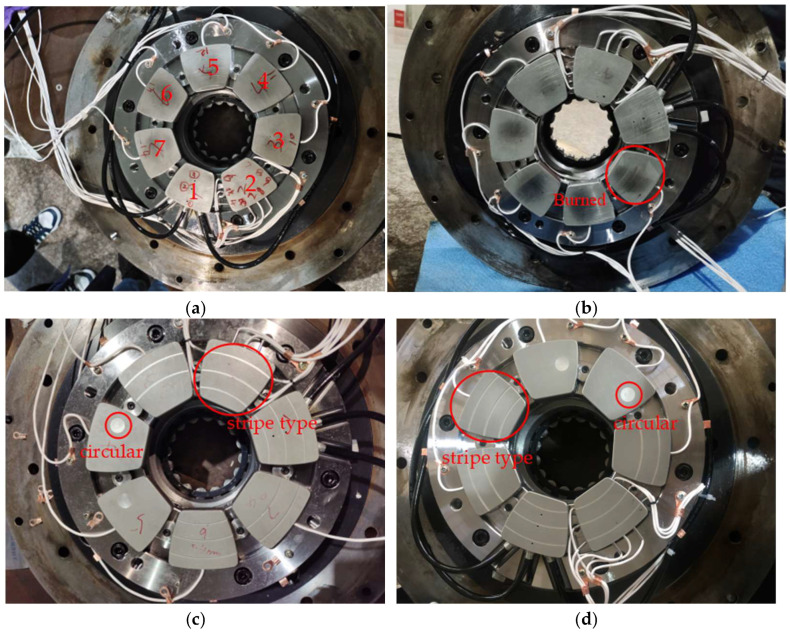
States of the tilting pad thrust bearing pads: (**a**) Normal; (**b**) Burned; (**c**) 2 mm scratched; (**d**) 3 mm scratched.

The experimental configuration involves specific states of the tilting pad thrust bearing pads, as shown in Figure 7. Various types of scratch damage were meticulously machined onto the pads, including stripe-type scratches of 2 mm and 3 mm in width, as well as circular scratches of the same dimensions. The spatial distribution of these scratches across the pads is visually shown in Figure 7, which correlates each scratch type with its respective pad position. For the 2 mm and 3 mm stripe scratches, three uniformly distributed scratches were introduced on pads No. 1, No. 2, and No. 6. Additionally, two scratches were applied to pads No. 3 and No. 7. The circular scratches were positioned to the right of the center on pads No. 4 and No. 5. Given that pad failure is processed manually, it is important to recognize that the actual failure mode may differ from expected scenarios. This discrepancy can introduce deviations in the acquired AE signals, potentially affecting the accuracy and reliability of the signal analysis. To mitigate biases in future studies, it is essential to maintain the equipment in operation until the pads fail.

**Figure 8:**
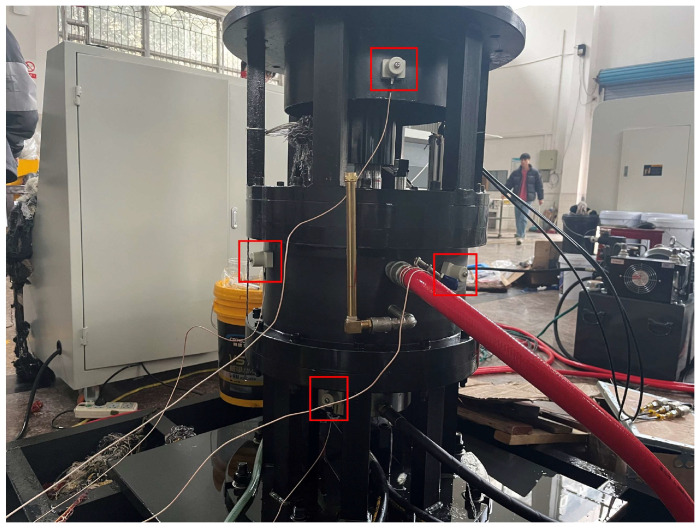
Arrangement of sensors in the experiment.

During the operation of the test bench, various sources generate AE signals beyond those originating from the tilting pad thrust bearing itself. These additional sources include AE signals from the two supporting rolling bearings, the coupling and shaft connections of the drive motor, and the oil pump inlet. The intermingling of these signals with those from the target object complicates the diagnostic process. Furthermore, the tilting pad thrust bearing operates within a lubricating oil environment, which prevents direct contact between the AE sensors and the sliding bearing surface. These factors collectively contribute to the complexity of the collected AE signals, thereby elevating the challenges associated with diagnostics. To mitigate these issues, seven AE sensors were deployed during the test. The sensors were positioned at various critical locations: one sensor on the motor guard, two sensors at the lubricant inlet and outlet of the installing box, two sensors on the side of the installing box, and two sensors on the outer ring surface of the spherical roller thrust bearing. The sensor arrangement is shown in Figure 8, with an additional three sensors positioned on the unshown side of the device.

## 4. Tilting Pad Thrust Bearing Fault Diagnosis Based on MMFCNN

### 4.1. Data Preprocessing

For comprehensive diagnostic analysis, the acquired AE signals are directly input into the subsequent CNN. This process requires the transformation of one-dimensional time-domain data into two- or multi-dimensional images. The pixel values of a grayscale image range from 0 to 255, with different values within this range representing different shades of gray. Utilizing this characteristic, the normalization and adjustment of the one-dimensional time-domain signal data are performed according to the following Equation (1):

(1) $$Y_{t}=255\frac{\left(X_{i}-X_{\min}\right)}{\left(X_{\max}-X_{\min}\right)}$$

where *X*_max_ is the maximum value in the AE data set, *X*_min_ is the minimum value in the AE data set, and *X_i_* is the *i*th original AE data.

**Figure 9:**
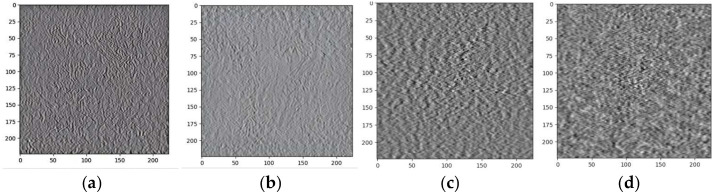
The grayscale representations of the four pad states: (**a**) Normal; (**b**) Burned; (**c**) 2 mm scratched; (**d**) 3 mm scratched.

The grayscale representations of AE signals corresponding to four distinct pad states are shown in Figure 9. Notably, these images exhibit substantial similarities across the different pad states, posing significant challenges for differentiation through visual inspection alone. Consequently, the application of advanced methodologies such as DL, particularly CNN, becomes crucial and effective. By leveraging these techniques, the model can learn and classify the features of the images, thus facilitating intelligent diagnosis and discrimination among the various pad states based on the collected AE signals.

### 4.2. Multi-Feature Fusion of Data

**Figure 10:**
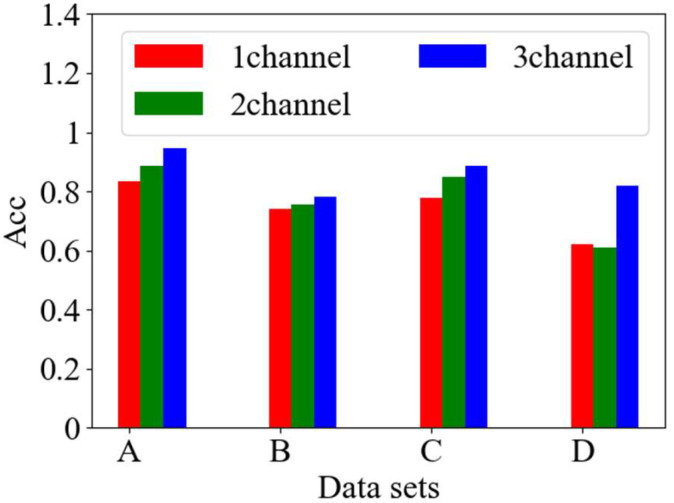
Accuracy of different channel models.

To validate the efficacy of the proposed feature fusion methods, the investigation focuses on the AE signal data obtained from tilting pad thrust bearings operating under various conditions, as shown in Table 3. The study evaluates the 1-channel model without feature splicing, the 2-channel model utilizing spanning convolutional feature splicing, and the 3-channel model that integrates three distinct features concurrently. The performance of the MMFCNN model is subsequently assessed across different input datasets, with results shown in Figure 10.

As shown in Figure 10, the 3-channel model, which incorporates simultaneous splicing of three features, exhibits superior diagnostic performance across the various datasets compared to the other models. The 2-channel model, utilizing spanning convolutional feature splicing, shows enhanced performance relative to the 1-channel model on the initial three datasets; however, it demonstrates inferior performance compared to the 1-channel model on the final dataset. Overall, the model that leverages fused multi-channel features captures a more comprehensive range of feature information than its counterparts, resulting in improved recognition outcomes across diverse datasets.

### 4.3. Comparison of the Performance of Different Networks

**Figure 11:**
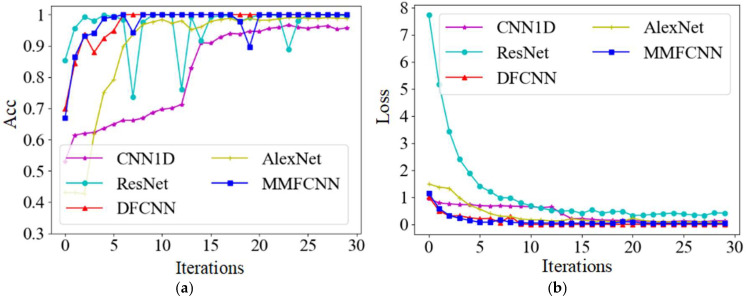
Diagnostic results of five network models: (**a**) Accuracy of the validation set; (**b**) Loss values for the training set.

The 1-dimensional convolutional neural network (CNN1D) [39] represent a specialized model adept at processing one-dimensional time series data. The residual neural network (ResNet) [40], a focal point in DL research, is renowned for its prowess in image recognition, enabling the extraction and classification of intricate elements within images to enhance accuracy. The deep feature convolutional neural network (DFCNN) [41] excels in extracting extensive and intricate features, thereby overcoming the limitations of conventional CNN in feature extraction. To evaluate the efficacy of the proposed model in this paper for fault diagnosis in tilting pad thrust bearings, a comparative experiment is conducted with four established models: CNN1D, ResNet, DFCNN, and AlexNet. AE signal data from tilting pad thrust bearings in four distinct states—normal, burned, 2 mm scratched, and 3 mm scratched—are utilized as inputs for training the aforementioned models. Each state comprises 1260 grayscale images at a scale of 224, totaling 5040 inputs. These inputs are partitioned into training, validation, and test sets in a ratio of 7:2:1. The activation function plays a crucial role in providing the nonlinear representational capabilities of a neural network. The rectified linear unit (ReLU) is widely used in CNN due to its speed and effectiveness in addressing the vanishing gradient problem, thanks to its linear and unsaturated characteristics [42]. The selection of hyperparameters (HPs) significantly influences the performance of neural networks. Even minor adjustments can lead to substantial advantages or disadvantages in the results. Therefore, the proper setting of HPs plays a crucial role in the effectiveness of fault diagnosis. Prior to the experiment, several preliminary tests were conducted to evaluate the effects of learning rate and batch size on network performance. The learning rate varied within the range of [0.0001, 0.01], while the batch size ranged from [2, 256]. Based on these tests, a learning rate of 0.001 and a batch size of 16 were identified as optimal for all participating networks. To ensure the reliability of the test results, the network parameters for each model used in the experiments were maintained consistently in accordance with those specified for the MMFCNN, as detailed in Table 2. Each model underwent 30 iterations during the testing phase, employing the ReLU activation function and stochastic gradient descent with momentum (SGDM) as the optimizer. The accuracy of the validation set for the five different networks and the loss of the training set are shown in Figure 11.

As shown in Figure 11a, the validation set accuracy of all five network models can exceed 90% after numerous iterations. Initially, ResNet achieves around 85% accuracy at the start of the iteration, but as the iteration count increases, its accuracy exhibits significant fluctuations, dropping to nearly 73.6% at one point. Throughout the training process, CNN1D consistently enhances its accuracy, with a notable variation occurring around the 13th iteration, followed by a gradual improvement and stabilization at approximately 95.7%. DFCNN and MMFCNN demonstrate higher accuracy levels while also maintaining better stability. Moreover, Figure 11b highlights the gradual reduction and eventual convergence of the training set loss for all five network models as the iterations progress.

**Figure 12:**
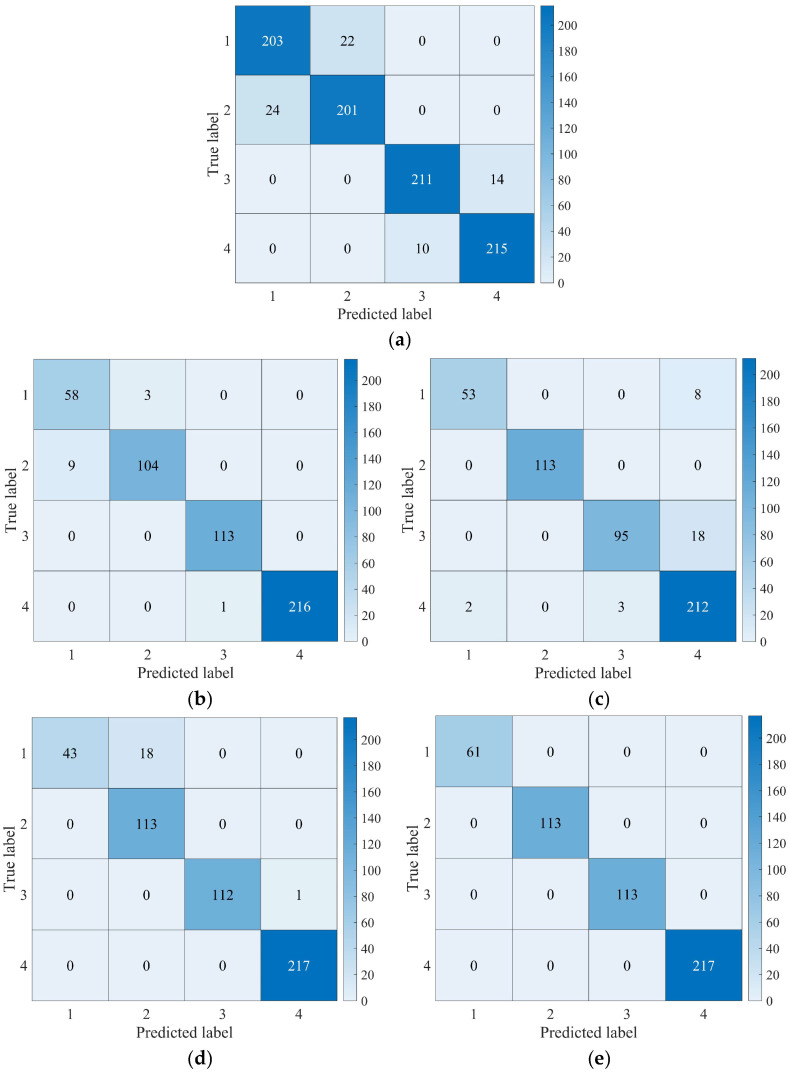
Confusion matrix of five network models test set: (**a**) CNN1D; (**b**) ResNet; (**c**) DFCNN; (**d**) AlexNet; (**e**) MMFCNN.

The parameters, including fixed weight bias, were maintained constant throughout the experiments. Validation of the model was conducted using test set data extracted from the preceding dataset. The confusion matrix corresponding to the test set of the five network models is shown in Figure 12.

To enhance the reliability of the experimental outcomes, the experiment was repeated 10 times, and the conclusive results for the test sets of the various network models are presented in Table 4.

The analysis of Figure 12 and Table 4 demonstrates that the average accuracy of the test sets across all five models exceeded 90%. Notably, the MMFCNN model exhibited the highest average accuracy among the models, achieving 99.58%, with a peak accuracy of 100%. In comparison, the CNN1D model exhibited the lowest average accuracy at 92.22%. The results highlight the effectiveness of DL approaches in accurately diagnosing fault types in tilting pad thrust bearings. Furthermore, in terms of standard deviation, MMFCNN exhibited significantly lower variability compared to the other models, indicating superior smoothness in its performance. This suggests that the MMFCNN model maintains consistent recognition accuracy levels with minimal fluctuation, enhancing its reliability for fault diagnosis applications. In terms of computational efficiency, the average computation time of the MMFCNN is 516s, which is lower than that of ResNet and DFCNN. However, CNN1D and AlexNet demonstrate superior computational efficiency compared to MMFCNN, primarily due to the simpler network architectures. In conclusion, MMFCNN exhibits commendable performance in terms of average accuracy, standard deviation, and average running time, and outperforms the other comparative models in general.

**Figure 13:**
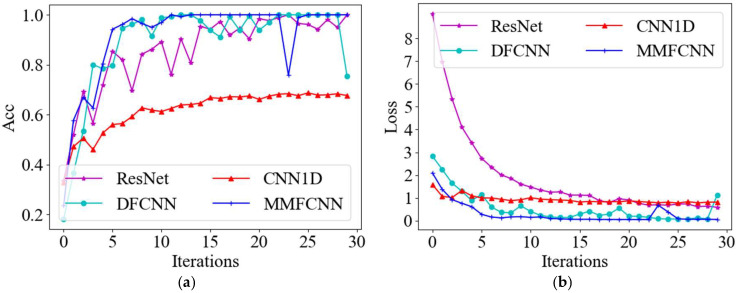
Diagnostic results of four network models: (**a**) Accuracy of the validation set; (**b**) Loss values for the training set.

In the operational context of tilting pad thrust bearings, variations in rotational speed, load, and other operational parameters lead to significant fluctuations in performance. These changes in operational conditions affect the AE signals generated by the bearings, introducing pronounced time-varying and non-linear characteristics. To investigate the effects of speed and load on the AE signal data of the tilting pad thrust bearing, a series of tests were conducted. The AE signal data were collected for two distinct speed ranges: 2900 rpm to 3000 rpm and 1400 rpm to 1500 rpm. Additionally, two load ranges were examined: 1 kN to 2.6 kN and 15.5 kN to 16.7 kN. All experiments were performed under controlled conditions at a constant temperature of 60 °C, and the data sets are shown in Table 5. The AE signal data, as outlined in Table 5, were utilized to evaluate the diagnostic performance of the model across various fault states of the tilting pad thrust bearings. The validation set accuracy and training set loss for the four distinct network models are shown in Figure 13.

**Figure 14:**
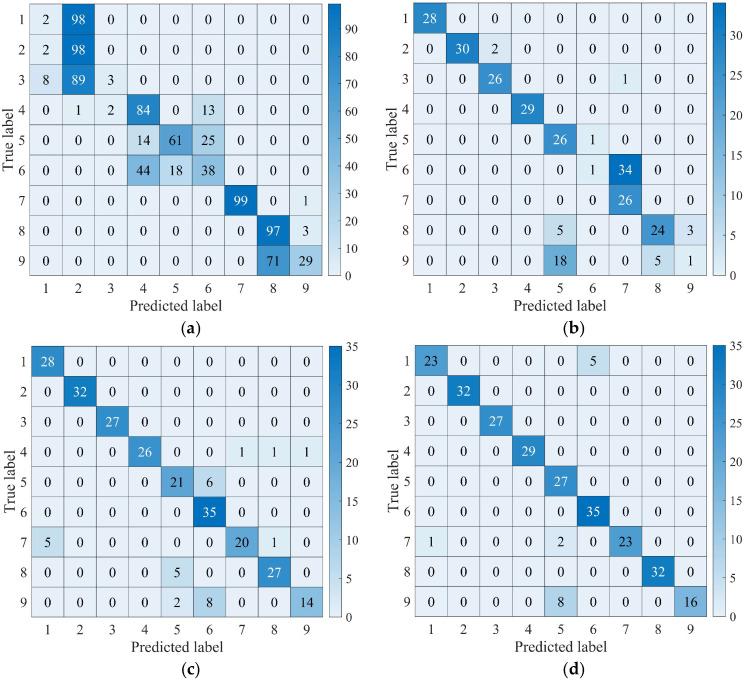
Confusion matrix of four network models test set: (**a**) CNN1D; (**b**) ResNet; (**c**) DFCNN; (**d**) MMFCNN.

As shown in Figure 13a, With the progression of iterations, CNN1D attains a recognition rate of approximately 67.7%. ResNet and DFCNN exhibit fluctuating accuracy rates, with DFCNN demonstrating superior diagnostic performance, generally surpassing ResNet in recognition rates. MMFCNN, on the whole, displays enhanced stability compared to other networks and tends to converge more rapidly. Moreover, Figure 13b highlights the gradual reduction and eventual convergence of the training set loss for all four network models as the iterations progress. Subsequently, the test set data is utilized to evaluate the four models, and the confusion matrix results are shown in Figure 14.

To enhance the reliability of the experimental outcomes, the experiment was repeated 10 times, and the conclusive results for the test sets of the various network models are presented in Table 6.

As shown in Figure 14 and Table 6, the integration of diverse working condition datasets resulted in a notable decrease in the average accuracy of the test sets for the four models when compared to the original datasets. Among the models evaluated, CNN1D achieved the lowest average accuracy at 56.44%. This may be attributed to the fact that variations in experimental rotational speed and loading do not significantly impact the AE signals produced by tilting pad thrust bearings, resulting in minimal differences in the input 1D time-domain signals. In contrast, both ResNet and DFCNN exhibited reduced accuracies compared to their performance without the dataset expansion. This decline is likely due to the grayscale maps being directly derived from the normalized 1D AE signal data. The grayscale maps generated from these one-dimensional signals lack distinct contrasting features, which hampers their effectiveness. On the other hand, even though the extended dataset does not introduce substantial differences in the 1D AE signals, the grayscale maps created through multichannel fusion remain more pronounced. Consequently, the MMFCNN model achieved an average accuracy of 93.51%, demonstrating outstanding fault diagnosis capabilities. Furthermore, the MMFCNN demonstrates the lowest standard deviation and better computational efficiency, with an average computation time that is only lower than that of the simpler 1DCNN architecture. This indicates that the MMFCNN maintains robust adaptability under varying operational conditions and when expanding the dataset.

### 4.4. Visualisation of the Model Learning Process

#### 4.4.1. t-Sne Visualization

**Figure 15:**
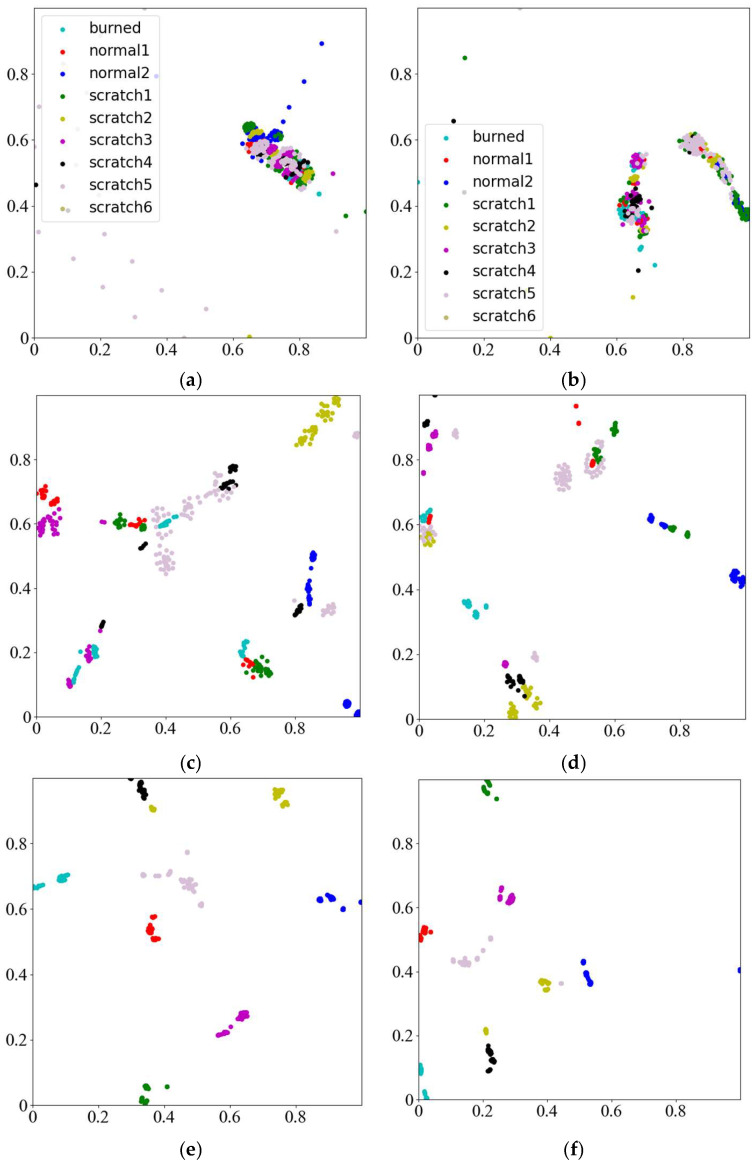
t-Sne visualization of individual layers of the MMFCNN model: (**a**) Convolutional layer 1; (**b**) Convolutional layer 2; (**c**) Convolutional layer 3; (**d**) Convolutional layer 4; (**e**) Convolutional layer 5; (**f**) full connectivity layer.

To gain deeper insights into the learning process of the model for subsequent optimization and refinement, the information acquired from various layers of the model is visualized using t-distributed stochastic neighbor embedding (t-SNE), utilizing the dataset derived from the tilting pad thrust bearing data outlined in Table 5. Figure 15 shows the t-SNE visualization of individual convolutional layers of the MMFCNN alongside the fully connected layers, offering a comprehensive overview of the model’s feature representations across different network depths.

As shown in Figure 15, there are discernible patterns as the model advances through its convolutional layers. Initially, after the first convolution, the categories cluster closely together with limited separability, accompanied by a few outliers that deviate significantly from the main distribution. Following the second convolution, the categories begin to diverge into two distinct blocks. Progressing through the third and fourth convolutions, the categories become more clearly separated, showing minimal large-scale mixing, although some overlaps persist between specific features such as scratch group 4 and scratch group 6 in the lower section of Figure 15d, as well as between scratch group 5 and burned group on the left. By the fifth convolution, the features exhibit a predominantly linearly separable structure, with each category occupying distinct regions in the feature space. The fully connected layer, which incorporates a substantial number of parameters, integrates all features, resulting in profound transformations characterized by significant shifts in individual feature positions and reduced distances among features.

Overall, the stacking of convolutional layers enhances the network’s capacity for feature expression. This progressive improvement facilitates effective category separation, underscoring the MMFCNN model’s proficiency in addressing the classification task involving nine distinct categories.

#### 4.4.2. Classification Process Feature Map Visualization

**Figure 16:**
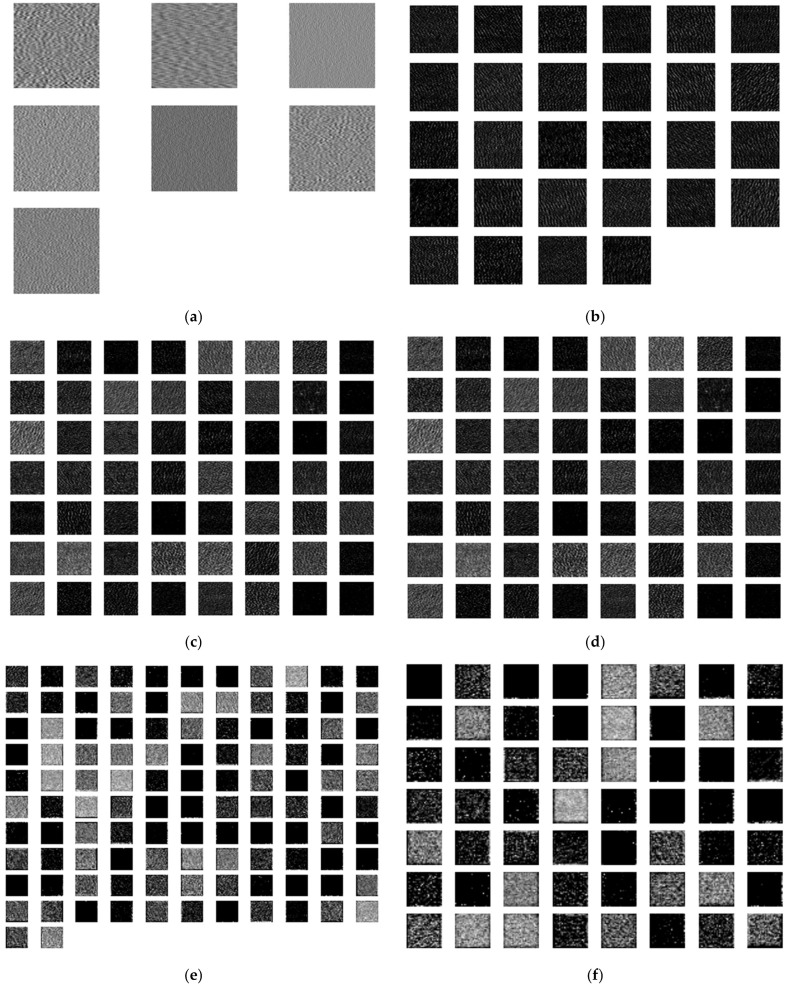
MMFCNN visualization of individual layers of grayscale images: (**a**) input layer; (**b**) Convolutional layer 1; (**c**) Convolutional layer 2; (**d**) Convolutional layer 3; (**e**) Convolutional layer 4; (**f**) Convolutional layer 5.

To gain deeper insights into the data transformations across various layers and channels during the network learning process. Taking the grayscale images of the pad fault type as burned as an example, Figure 16 shows the changes in the grayscale images at the input layer and in the subsequent convolutional layers (1–5) after the data has been processed by the MMFCNN model.

**Figure 17:**
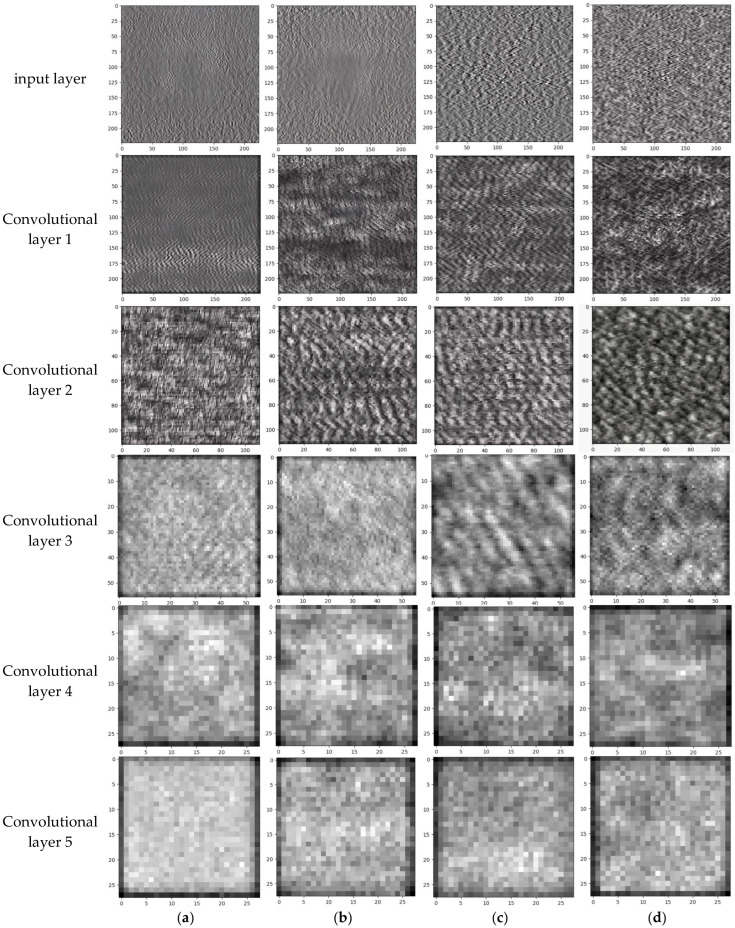
Visualization of fault characteristics at individual layers of MMFCNN: (**a**) Normal; (**b**) Burned; (**c**) 2 mm scratched; (**d**) 3 mm scratched.

After inputting the AE signal data of the tiltable tile thrust bearing in four distinct operational states into the MMFCNN, the grayscale images corresponding to the input layer and each convolutional layer are combined in a 1:1 ratio. This fusion process yields an overall grayscale image representing the output of each layer, as shown in Figure 17.

As shown in Figure 17, there exists a significant disparity between the grayscale image generated after traversing five convolutional layers and the original grayscale input image. Particularly, the grayscale maps in the initial three convolutional layers can distinctly differentiate differences across different states.

## 5. Conclusions

(1) A multi-feature fusion convolutional neural network (MMFCNN) is proposed in this study. The network comprises three feature extraction channels aimed at capturing dataset features from diverse perspectives. Subsequent to feature extraction, fusion from the depth direction is achieved through feature splicing after the pooling layer. Comparative analysis is conducted on models with varying numbers of channels, revealing that the inclusion of both auxiliary channels simultaneously leads to improved results across different datasets.
(2) Focusing on the AE signals from four states of tilting pad thrust bearings, the proposed MMFCNN network model is compared and tested against traditional network models such as CNN1D, ResNet, DFCNN, and AlexNet. The diagnostic performance of the models is evaluated based on criteria including recognition rates during training and validation stages, stability, convergence speed, and accuracy on the test set. Experimental results demonstrate that the MMFCNN outperforms other models across all metrics, achieving an average test set accuracy of 99.58%.
(3) To investigate the impact of rotational speed and load variations on the AE signals of tilting pad thrust bearings, experiments were conducted by altering the rotational speed and load while maintaining a constant ambient temperature. A dataset comprising nine data types from four states of tilting pad thrust bearings was collected for analysis. The proposed model was compared with CNN1D, ResNet, and DFCNN models using this dataset. Results indicate that even with the dataset expansion, MMFCNN continues to exhibit superior performance compared to the other models.

This study successfully diagnoses tilting pad thrust bearings using AE signals and CNN. However, the application of this methodology in industrial systems remains challenging. The failures of tilting pad thrust bearings often exhibit significant diversity and complexity, complicating the acquisition of sufficient data for effective analysis. On the one hand, a real-time monitoring system can be developed to collect operational data from tilting pad thrust bearings. This system would not only facilitate the continuous monitoring of performance but also assist in the early detection of potential failure signs, thereby ensuring the safe operation of equipment. On the other hand, the implementation of remote monitoring technology can enable the timely transmission of the collected data to a centralized server or analysis platform, allowing for prompt data evaluation. However, practical implementation faces difficulties due to the challenges associated with sensor placement and the complex spatial arrangement of tilting pad thrust bearings within machinery. To overcome these obstacles, future research could investigate the deployment of non-contact sensors, which would simplify data acquisition processes. Furthermore, the integration of machine learning and artificial intelligence techniques could enhance data augmentation and facilitate intelligent analysis of limited datasets, thereby improving diagnostic capabilities.

Considering the limitations of the acquired dataset presented in this study, as well as the fact that the proposed method is validated using only four types of pads, in the future, we will further expand the dataset and apply the proposed method to additional types of plain bearing. Furthermore, we will also make further improvements to the MMFCNN model to improve its fault diagnosis capabilities.

## Figures and Tables

**Table 1:** MMFCNN model parameters.

Layer	Type	Kernel Size	Stride	Channel
0	Input	-	-	7
1	Convolution 1	11, 2, 113	1, 2, 1	28, 14, 7
2	Pooling	2	2	28
3	Convolution 2	5, 4, 57	1, 4, 1	56, 28, 7
4	Pooling	2	2	56
5	Convolution 2	5, 8, 29	1, 8, 1	112, 56, 7
6	Pooling	2	2	112
7	Convolution 3	3	1	112
8	Convolution 4	3	1	56
9	Fully connected	-	-	2048
10	Convolution 5	-	-	2048
11	Fully connected	-	-	4

**Table 2:** Fault category and description.

Group	Fault Type	Fault Description
1	Normal	Damage-free
2	Burned	Burned after start/stop with load
3	2 mm scratched	Processed with 2 mm scratches manually
4	3 mm scratched	Processed with 3 mm scratches manually

**Table 3:** Data set of tilting pad thrust bearings for various conditions.

Data Set	Load/kN	Temperature/°C	Speed/rpm	Fault Type
A	16.7	60	2970	Normal, Burned, 2 mm scratched, 3 mm scratched
B	11.5–20	65	1485	2 mm scratched
C	16.7	45–60	2970	3 mm scratched
D	A, B, C			

**Table 4:** Results of five network model test sets.

Test Model	Average Accuracy (%)	Standard Deviation	Average Computation Time (s)
CNN1D	92.22	0.6241	358
ResNet	95.64	0.9940	1733
DFCNN	94.97	0.7542	584
AlexNet	94.78	0.9451	478
MMFCNN	99.58	0.3295	478

**Table 5:** Data set of tilting pad thrust bearings with diverse speeds and loads.

Group	Fault Type	Speed (rpm)	Load (kN)
1	Normal	2900–3000	15.5–16.7
2	Burned	2900–3000	15.5–16.7
3	2 mm scratched	2900–3000	15.5–16.7
4	3 mm scratched	2900–3000	15.5–16.7
5	Normal	2900–3000	1–2.6
6	2 mm scratched	1400–1500	15.5–16.7
7	2 mm scratched	2900–3000	1–2.6
8	3 mm scratched	1400–1500	15.5–16.7
9	3 mm scratched	2900–3000	1–2.6

**Table 6:** Results of four network model test sets.

Test Model	Average Accuracy (%)	Standard Deviation	Average Computation Time
CNN1D	56.44	0.4769	316
ResNet	72.65	0.9333	1042
DFCNN	88.08	0.8122	512
MMFCNN	93.51	0.4249	435

## Data Availability

Data are unavailable due to privacy restrictions.
